# Neuroplasticity Regulation by Noradrenaline in Mammalian Brain

**DOI:** 10.2174/157015909790031193

**Published:** 2009-12

**Authors:** Aude Marzo, Jing Bai, Satoru Otani

**Affiliations:** 1 INSERM UMRS 952, 9 Quai St Bernard, 75005, Paris, France; 2 CNRS UMR 7224, 9 Quai St Bernard, 75005, Paris, France; 3 Université Pierre et Marie Curie (UPMC)-Paris 6, 75005 Paris, France; 4 Sophia University Open Research Center, Tokyo, Japan

**Keywords:** Noradrenaline, LTP, LTD, neuromodulation, synaptic plasticity.

## Abstract

The neuromodulator noradrenaline (NA) is released in almost all brain areas in a highly diffused manner. Its action is slow, as it acts through G protein-coupled receptors, but its wide release in the brain makes NA a crucial regulator for various fundamental brain functions such as arousal, attention and memory processes [102]. To understand how NA acts in the brain to promote such diverse actions, it is necessary to dissect the cellular actions of NA at the level of single neurons as well as at the level of neuronal networks. In the present article, we will provide a compact review of the main literatures concerning the NA actions on neuroplasticity processes. Depending on which subtype of adrenoceptor is activated, NA differently affects intrinsic membrane properties of postsynaptic neurons and synaptic plasticity. For example, β-adrenoceptor activation is mainly related to the potentiation of synaptic responses and learning and memory processes. α2-adrenoceptor activation may contribute to a high-order information processing such as executive function, but currently the direction of synaptic plasticity modification by α2-adrenoceptors has not been clearly determined. The activation of α1-adrenoceptors appears to mainly induce synaptic depression in the brain. But its physiological roles are still unclear: while its activation has been described as beneficial for cognitive functions, it may also exert detrimental effects in some brain structures such as the prefrontal cortex.

## INTRODUCTION

Neuroplasticity can be defined as the potential of neural elements to react with adaptive changes to intrinsic or extrinsic inputs. It is the principal flexible property of neurons, or rather neuronal networks, through which they temporarily or permanently change their biochemical, physiological and morphological characteristics. These characteristics make neuroplasticity a good candidate for the basis of learning and memory.

Classically, NA is thought to act on three main classes of plasticity changes in the nervous system, which are the developmental (which we shall not address in this article), neuronal (or intrinsic), and synaptic plasticity. The literature highlights two points that 1) NA acts on the excitability of neurons, and that 2) NA modulates synaptic plasticity as well as itself inducing synaptic plasticity.

In the present article, we will first briefly introduce the NA system and adrenoceptor-related intracellular pathways. Second, we will review the literatures on how the stimulation of adrenoceptors modulates cellular excitability (i.e. neuronal/intrinsic plasticity). Then, we will introduce studies on short-term and long-term synaptic plasticity, which are induced or modulated by NA. Finally, the putative functional relevance of these plasticity processes will be briefly discussed.

## NORADRENERGIC SYSTEM

NA is released in the entire brain areas with the exception of the basal ganglia, from the locus coeruleus (LC), the bilateral small nuclei located in the dorsal tegmentum. Cortical NA innervation is described as mostly non-synaptic, which may support the evenly diffused release of cortical NA to the extracellular space, compatible with both its neuromodulatory role and the multiplicity of its actions on diverse cellular targets in the cerebral cortex [111]. A single LC neuron may project to both cortical hemispheres of different cortical areas [34]. This way, the LC may innervate functionally diverse target areas simultaneously with a global and uniform activity. This may be one way in which the LC coordinates the activity of multiple brain systems [124].

There are three subtypes of adrenoceptors, β, α_1_ and α_2_ [134], which are all known to be metabotropic receptors, i.e. a class of receptors linked to G protein. The affinity of NA is higher for α_2_- than for α_1_-receptors, and both of these receptors have higher affinities to NA than β-receptors [8].

### β-Adrenoceptors

Three types of β-receptors are described in the brain, i.e. β_1_-, β_2_- and β_3_-adrenoceptors [98, 118]. To date, these three β-receptors are not very well distinguished, since specific agonists/antagonists have not been extensively used in preceding studies. 

The β-receptors are mainly located in postsynaptic neurons, although a small proportion may exist in presynaptic components in some regions such as the dentate gyrus [80] and prefrontal cortex (PFC) [50]. These receptors are associated with the activation of Gs that activates adenylate cyclase and produces cyclic adenosine monophosphate (cAMP), which can be further associated with CREB (cAMP response element-binding) protein activation. The three β-adrenoceptor subtypes are subject to a desensitization by means of uncoupling from their G proteins, the process being governed by G protein-coupled Receptor directed Kinases (GRKs) as well as by specific kinase like β-adrenergic receptor kinase (β-ARK; [24]).

### α_1_-Adrenoceptors

α_1_-adrenoceptors are postsynaptic receptors and composed of three subtypes: α_1A_, α_1B_ and α_1D_. These three subtypes are equally expressed in the hippocampus, the cerebral cortex and the brainstem, but in the thalamus and deep layers of parieto-frontal cortex, α_1A_-adrenoceptors are preferentially expressed [53, 94].

All three subtypes (α1A-, α1B- and α1D-adrenoceptor) are able to mobilize calcium ions from intracellular stores as well as to increase the calcium entry *via* voltage-gated calcium channels. Stimulation of all three α1-adrenoceptor subtypes leads to the hydrolysis of membrane phospholipids *via* G protein-mediated activation (Gq protein) of phospholipase Cβ. The resultant production of inositol triphosphate (IP3) mediates the α1-adrenoceptor-elicited calcium release from intracellular stores, thereby increasing cytosolic calcium concentrations. The simultaneously produced diacylglycerol (DAG) activates protein kinase C (PKC) [120], which is also activated by a group of calcium and calmodulin-sensitive protein kinases. Active PKC phosphorylates many cellular substrates including membrane channels, pumps, and ion-exchange proteins. The α1-adrenoceptors also have been reported to modulate other signaling pathways: their activation may result in an increased accumulation of cAMP and cGMP, a potentiation of cAMP responses elicited by Gs-linked receptors [51], the activation of phospholipase A2 and phospholipase D, the activation of cAMP phosphodiesterase, release of adenosine, and the stimulation of arachidonic acid release [134]. This class of receptors is also subject to desensitization by GRKs [97].

### α_2_-Adrenoceptors

Three subtypes of α_2_-adrenoceptors are described, known as α_2A_-, α_2B_-, and α_2C_-adrenoceptors. Their mRNA shows a widespread distribution in the brain and is expressed primarily in regions of the brainstem that contain adrenaline- and NA-producing cells, but is also expressed in several other areas including the hippocampus and the cerebral cortex [90, 108]. PFC neurons express principally the α_2A_-subtype [6].

α_2_-adrenoceptors are located on both pre- and postsynaptic sites. The presynaptic localization indicates their functions as autoceptors, involved in the control of NA release by LC neuronal axons. Sub-cellularly, α_2A_-adrenoceptors in the LC and PFC are associated with synaptic and non-synaptic dendritic and perikarya membranes [5]. In addition, the cortical neurons, but not LC neurons, exhibit prominent immunoreactivity to α_2A_-adrenoceptors within dendritic spine heads [5].

α_2_-adrenoceptors are classically linked to Gi/o protein whose action is opposite to that of Gs. These receptors act through inhibiting adenylate cyclase *via* Gi protein and thereby inhibit the production of cAMP, while the βγ subunits of Gi protein increase potassium ion conductance. α_2_-adrenoceptors also suppress voltage-activated calcium channels *via* Go proteins, thus reducing the flow of extracellular calcium ions into target cells. Moreover, growing lines of evidence suggest that α_2_-adrenoceptors are linked not only to the activation of Gi/o cascade but also, for example, the activation of phospholipase C (PLC) and PKC at least in some cell types [20, 119]. As in the case of the other two classes of receptors, α_2_-adrenoceptors can be desensitized by GRKs, resulting in a functional uncoupling from their G-proteins [134].

## INTRINSIC PLASTICITY

**Fig. (1). Schematic representations of the action of adrenoceptors that induce intrinsic plasticity.**
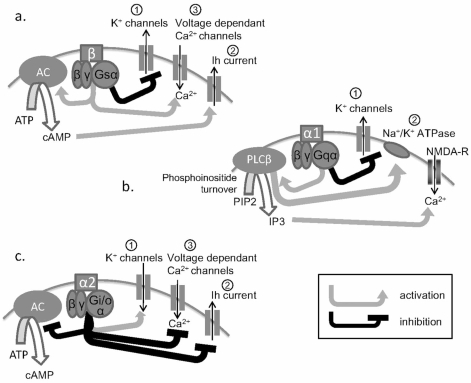
**a.** β-adrenoceptor activation generally induces depolarization of postsynaptic neurons with an increase of input resistance. It reduces K^+^ currents (1) and facilitates *Ih* current via cAMP pathway (2) and the entry of Ca^2+^ (3). **b.** α_1_-adrenoceptors can also induce depolarization of neurons with an increase of input resistance. They reduce K^+^ currents (1) and act on Ca^2+^ entry via the activation of phosphoinositide turnover (2). **c.** α_2_-adrenoceptors generally induce hyperpolarization coupled to an increase or decrease of input resistance via blockade of *Ih* current (2) or opening of K^+^ channels (1) respectively. They also inhibit Ca^2+^ channels (3). AC, adenylate cyclase; ATP, adenosine triphosphate; Ca^2+^, calcium; cAMP, cyclic adenosine monophosphate; DAG, diacylglyrerol; *Ih*, hyperpolarization-activated currents; IP3, inositol (1,4,5)-trisphosphate; K^+^, potassium; PIP2, phosphatidylinositol biphosphate; PLCβ, phospholipase Cβ.

By the term neuronal or intrinsic plasticity, we shall refer to adaptive alterations of postsynaptic excitability, which are non-synaptic in nature and thus mechanistically internal to a given postsynaptic cell. Thus, NA activation of postsynaptic adrenoceptors results in the activation of various intracellular factors and triggers for example modifications of membrane ion channel properties (Fig. **1**). This type of plasticity is crucial for neuronal function given that it directly modulates cellular characteristics such as ion channel opening. This class of plasticity may be temporally short-lived (observed only in the presence of agonists, for example) or may be long-lasting (observed even well after washout of the agonists or other induction agents or events). Importantly, as mentioned in the next section (see “Synaptic plasticity induced by NA”), increases in postsynaptic excitability through the induction of intrinsic plasticity, particularly after β-adrenoceptor activation, may constitute the mechanistic basis for long-lasting potentiation of the population spike. However, this potentiation is detected by means of synaptic stimulation. We will therefore list this potentiation under the next synaptic plasticity section.

### β-Adrenoceptor Cellular Effects

The activation of β-adrenoceptors acts on three cationic currents and induces intrinsic plasticity (Fig. **1a**).

1. It may decrease the potassium conductance [41]. This effect results in a depolarization of postsynaptic membrane usually associated with an increase in the input resistance as shown in rat CA1 pyramidal neurons [65, 66] and layer II-III neurons of frontal cortex [28] (but see [76] for a decrease of input resistance in the thalamus). Potassium conductance can also be decreased through, for example, a block of calcium-dependent potassium channels as demonstrated in the hippocampus (dentate gyrus and CA1) [42].
  This inhibition of potassium conductance may underlie the blockade of the slow after-hyperpolarization (AHP) current in the cortex [33], the thalamus (paratenial thalamic nucleus; [75, 76]), and the hippocampus [41, 42, 57, 65, 66].
2. β-adrenoceptor activation may enhance hyperpolarization-activated current (*I*h) that is carried by sodium and potassium ions, due to an increase of intracellular concentration of cAMP as shown in guinea-pig dorsolateral geniculate nucleus [75, 95] and rat thalamic neurons [136].
3. Finally, β-adrenoceptor activation allows the enhancement of certain voltage-dependent calcium currents in the dentate gyrus [40], hippocampal CA3 [32], and the PFC [50].

### α_1_-Adrenoceptor Cellular Effects

α1-adrenoceptors exert a general excitatory effect with a depolarization of resting membrane potential often associated with enhanced input resistance [2, 77, 93] (but see [99] for no change in input resistance) (Fig. **1b**).

1. α1-adrenoceptor activation decreases potassium currents [74, 76]. The particular types of potassium currents reduced by α1-adrenoceptors include *I*_A_ [2] and the leak potassium current (*I*_KL_) [77]. A decrease in the latter potassium current is associated with a change in the firing pattern of the neuron. A reduction of *I*_KL_ induces a shift of firing mode from rhythmic oscillation to tonic single spike activity in the thalamus in guinea pigs and cats [74, 75] (see [77] for review). A similar result was found in γ-aminobutyric acid (GABAergic) cells of the thalamus, where NA produces a prolonged increase of cellular excitability due to a slow depolarization, which is surprisingly accompanied by a decrease in the input conductance. α1-adrenoceptor activation also prolongs after-hyperpolarization in dorsal raphe neurons [37].
2. Besides these potassium current modulations, α1-adrenoceptors activation and their consequential augmentation of intracellular calcium concentration may potentiate the activation of Na/K-ATPase [69].

### α_2_-Adrenoceptor Cellular Effects

α_2_-adrenoceptors induce postsynaptic membrane hyperpolarization (Fig. **1c**; [115]).

1. This hyperpolarization can be associated with decreases in input resistance, which may derive from the modulation of potassium channels, in spinal cord [92], hippocampal CA1 pyramidal neurons [65], and neocortical neurons [28]. The hyperpolarization may also be associated with a decrease in cAMP levels as shown in LC neurons [4], or can be due to an opening of ATP-dependent potassium channels (K-ATP channels) by Gi/o protein [137].
2. α_2_-adrenoceptors have also been seen to induce hyperpolarization with increases in the input resistance in medial PFC. In this case, opposite to β-adrenoceptors, α_2_-adrenoceptors block hyperpolarization-activated currents (*I*h) [14] *via* blockade of hyperpolarization/cyclic nucleotide gated cation (HCN) channels. It is proposed that the overall effect of HCN channel inhibition is to suppress the response to isolated excitatory inputs while enhancing the response to a coherent burst of synaptic activity [20].

In addition, α_2_-adrenoceptor agonists inhibit voltage-activated calcium currents mediated by the N- or P-type calcium-channels [26, 122]. This suppression of voltage-sensitive calcium channels, as well as the inhibition of adenylate cyclase by Gi/o protein and the activation of potassium channels by α_2_-adrenoceptors, can all contribute to the reduction of neurotransmitter release [134].

## SYNAPTIC PLASTICITY INDUCED BY NA

As described above, NA is generally thought to belong to the category of neuromodulators, which signifies functionally that NA acts on the neurons through modifications of currently ongoing events. From this aspect, NA has been extensively studied in the field of synaptic plasticity. But some evidence suggests also that NA itself can initiate synaptic plasticity. The majority of studies have been conducted in the hippocampus and sensory cortical areas. The term “synaptic plasticity” signifies that modifications occur at the level of the synapse. In this sense, it may be directly related to, or independent of, the intrinsic plasticity described in the preceding section.

At present, two examples of synaptic plasticity are well-known and have been extensively studied; i.e. long-term potentiation (LTP) and long-term depression (LTD). LTP is commonly defined as a lasting increase of synaptic efficacy and has been observed often in glutamatergic synapses in various brain regions such as the hippocampus and the PFC [39, 68]. LTD is, in contrast, a lasting decrease of synaptic efficacy and also observed in glutamatergic synapses across many brain areas [39, 68]. Modulation of LTP/LTD and the induction of LTP/LTD-like changes by NA are discussed in this section.

In addition, in the 1970’s, an important trend emerged which stated that NA might enhance the “signal-to-noise ratio”, which is in close relation with synaptic plasticity. The pioneers in this field were Foote *et al*. [35] and Segal and Bloom [109] who worked on auditory cortical and hippocampal neurons, respectively. This denomination suffers from the weakness that the basal or background activity cannot be considered simply as “noise”.

### NA and Potentiation

A large part of the hippocampal literature describes the NA action of facilitating or inducing potentiation (i.e. LTP) through β-adrenoceptors. *In vivo* and *in vitro* studies conducted in the dentate gyrus and in area CA1 of the hippocampus show that the application of NA by itself induces a potentiation of the population spike, augmenting "E-S (EPSP-spike) coupling" (e.g. [23, 43, 88, 100, 133]). This effect seems to be mediated by β-adrenoceptors [43] and cAMP [30], and is likely to be mechanistically supported by the induction of intrinsic plasticity mentioned in the previous section [30]. This LTP of the population spike appears also to require concurrent synaptic activation of NMDA receptors [18], suggesting the existence of convergent action of β-adrenoceptors and NMDA receptors (but see [44] for NMDA receptor-independency of this NA effect). In the dentate gyrus *in vivo*, it was also shown that stimulation of β-adrenoceptors by endogenous NA induces LTP of the population spike [44, 84]. This NA-induced LTP was however shown to be mechanistically different from high-frequency stimulation-induced LTP of the population spike [58], although this latter tetanus-induced LTP depends on NMDA receptors [1].

NA also facilitates LTP induced by high-frequency afferent stimulation. This was shown in area CA3 [46] and dentate gyrus [3, 18]. This NA facilitation of LTP is achieved through β-adrenoceptors [47] and can be mimicked by an adenylate cyclase activator (i.e. through the increase of cAMP; [52]) similar to the aforementioned case of LTP of the population spike induced by bath-application of NA. These examples of cAMP involvement in NA-induced or NA-facilitated LTP are consistent with another report suggesting that β-adrenoceptor stimulation facilitates LTP in area CA1 through protein kinase A (PKA) activation [132]. This PKA involvement was shown to depend on the stimulus pattern used to induce LTP, where the β-adrenergic facilitation of LTP induced by 5 Hz stimulation involves PKA [38, 132] whereas a similar facilitation of LTP triggered by 100 Hz stimulation does not [38]. Similarly, in area CA1, associative LTP induced by low-frequency paired stimulation was facilitated by PKA activated through β-adrenoceptors [63]. These cases of PKA-dependent LTP facilitation were shown to involve the extracellular signal regulated kinase (ERK) pathway [63, 132].

Depending on the strength of tetanus used to induce LTP, β-adrenoceptors also modulate the maintenance of the late phase of LTP (L-LTP) in the dentate gyrus [116]. A more recent report proposes that NA facilitates LTP induction in the hippocampus by phosphorylation of the GluR1 subunit, which facilitates the delivery of GluRs into synapses [48]. In the visual cortex also, NA facilitates LTP through β-adrenoceptors stimulation, and in this case, NA acts synergistically with muscarinic acetylcholine receptors [17]. Since the visual cortex LTP is dependent also on NMDA receptors [11], this result again indicates the convergent action of multiple neurotransmitters for LTP. In the medial amygdala, short-term potentiation induced by high-frequency stimulation was shown to convert to LTP in the presence of β-adrenoceptor agonist [128]. Interestingly, in the study of Watanabe *et al*. described above [48], it was shown that, although LTP is induced by tetanic stimulation combined with a β-adrenoceptor agonist in the medial part of the amygdala, the same protocol suppressed normal short-term potentiation in the lateral amygdala [128]. Similarly in the dentate gyrus, LTP or LTD can be facilitated depending on whether responses are evoked by medial or lateral perforant pathway stimulation [22, 96].

α_1_-adrenoceptors have been, shown to enhance the frequency of excitatory postsynaptic currents (EPSCs) in medial PFC neurons [73]. This class of receptors also enables the augmentation of synaptic density in rat visual cortex, which might be essential for the maintenance of synapses as well as for synaptic plasticity [86]. Moreover, Segal *et al*. [110] showed the enhancement of responses to NMDA in the presence of a α1-agonist in CA1 hippocampal neurons, an action achieved *via* the activation of phosphoinositide turnover.

### NA and Depression

NA has also been described to induce synaptic depression. In transverse slices of rat visual cortex, it was shown that paired-pulse stimulation induces NMDA receptor-dependent acute and long-lasting homosynaptic depression in the presence of NA acting on α_1_-adrenoceptors [56]. A similar facilitation of synaptic depression was seen in the presence of acetylcholine [86]. These results parallel those reported by Scheiderer *et al*. [107] in the hippocampus, where muscarinic or adrenergic receptor activation induced LTD. In another study, Liu *et al*. [64] focused on NMDA receptor-mediated EPSCs in the PFC and showed that α_1_-adrenoceptor stimulation induces depression. We have also found that NA induces LTD of glutamatergic transmission in PFC slices *via* the activation of α_1_- and α_2_-adrenoceptors (Marzo *et al.*, unpublished data). The majority of the examples of synaptic depression induced by NA involves α_1_-adrenoceptors, but not β-adrenoceptors [64, 78, 106]. Indeed, α- and β-adrenoceptors appear to exert opposite effects on synaptic transmission, i.e. facilitation of LTD and LTP, respectively. This was further suggested by the fact that NA induces LTP in dentate gyrus when applied with a α-adrenoceptor antagonist [22].

The locus of the induction of LTD has been shown to be postsynaptic in the PFC (Marzo *et al.*, unpublished data). Also, the absence of changes in paired pulse facilitation supports the postsynaptic locus of induction for the acute depression of NMDA receptor-mediated synaptic responses in the PFC [64].

### NA Effects on Inhibitory Transmission

The majority of studies on the NA effects on synaptic transmission focused on excitatory glutamatergic transmission. However, NA also acts on GABAergic transmission. Generally, increases of synaptic inhibition by NA are noted. For example, in the frontal cortex, NA induces an increase in the frequency of inhibitory postsynaptic currents (IPSC) recorded from pyramidal neurons, with NA acting through the enhancement of excitability of GABAergic neurons *via* α-adrenoceptor stimulation [54]. Similarly in the entorhinal cortex, α1-adrenoceptor activation increases the frequency of miniature IPSCs, suggesting a presynaptic effect [60]. In area CA1 of the hippocampus, on the other hand, NA decreases inhibitory postsynaptic potentials *via* α-adrenoceptor activation [67]. In the somatosensory cortex, it was shown that NA enhances GABA-induced inhibition [129] which in this case is mediated by β-adrenoceptor-inducing augmentation of cAMP [114]. Similar results were found in lateral hypothalamus [113] and cerebellum [21, 81]. In cerebellum, α-adrenoceptors are also involved in the increase in the IPSC with distinct roles played by α1- and α2-adrenoceptors [45]: thus, α1-adrenoceptor activation increases the spontaneous and evoked IPSC, but α2-adrenoceptors rather decrease the spontaneous IPSC without affecting the evoked IPSC.

## FUNCTIONAL RELEVANCE OF NA INDUCED PLASTICITY

Different lines of evidence suggest that NA-induced plasticity may have multiple functional roles. For example, the induction of intrinsic plasticity would increase the probability of certain patterns or modes of neuronal discharge, and such changes may be related to the level of waking and arousal [12, 75]. NA system-related arousal in turn may participate in the information processing as indicated by the fact that LTP induction is modulated by appetitive and aversive stimuli [112]. In this respect, Seidenbecher *et al*. [112] showed a reinforcement of hippocampal LTP triggered by a sub-threshold tetanus in the presence of an event known to activate release of NA in the hippocampus. This LTP reinforcement was blocked by the administration of β-antagonist.

The crucial role of β-adrenoceptor activation for the maintenance of L-LTP [116] may be regarded as a basis for the consolidation of long-term memory. In fact, in behavioral studies, the NA system has been implicated in the consolidation and retrieval of memory [79, 101], partly from the effect of enhanced arousal [19]. It appears that β-adrenoceptors are the subtype involved in the memory consolidation, since, for example, β-adrenergic antagonists cause amnesia in spatial memory paradigms [101, 103]. β-adrenoceptors in the rat prelimbic area are also involved in a late phase of long-term olfactory memory consolidation [123]. Moreover, it has been suggested that memory consolidation is achieved by a long-term effect of NA on synaptic transmission, taking place during slow wave sleep (evidence demonstrates that the LC is transiently activated during this sleep phase after intensive learning [31]).

Retrieval is also an important step in memory processes during which NA appears to act. It was shown that mutant mice that cannot synthesize NA can still learn a contextual fear-conditioning task but are impaired in retention when tested 2 days later [85]. This retention deficit was rescued by the injection of a precursor of NA before the test, demonstrating that NA is necessary for the access to a memory trace at this time [85]. These results taken together suggest that NA is important for consolidation and retrieval of some types of memory. However, it should be pointed out also that in the case of amygdala-dependent fear memory, NA through β-adrenoceptors participates rather in memory re-consolidation but not its consolidation or retrieval [25, 85].

Another main function related to the NA system is the information processing from sensory collections to the high-order cognition [102]. Thus, NA has been shown to be crucial in tasks involving changes of strategy [82, 89], and this effect appears to depend on α-adrenoceptors. But there is still a controversy as to the role of α-adrenoceptors in cognitive functions [10]. For example, the activation of α1-adrenoceptors in the PFC is classically related to the impairment of cognitive performance [9], whereas α2-adrenoceptors are known to improve it [61]. Nevertheless, blockade of α1-adrenoceptors when NA levels are pharmacologically enhanced actually blocks the NA-induced enhancement of performance in the strategy switching [58].

Mechanistically, the above flexibility for strategy adoption may be related to LTD induction as shown in the hippocampus [29, 91]. It is thus possible that LTD generally weakens network efficacy so that it enables the neural network to select and encode new representations through the readily potentiable synapses.

Another line of evidence relates to novelty exploration. It is known that LC neurons show enhanced discharge in response to novel stimuli and rapidly habituate after a few encounters with the same stimuli [125]. This characteristic of LC neurons suggests that NA may participate in the process of information acquisition [13, 27, 104, 105], perhaps through the induction of synaptic plasticity. Both directions of synaptic plasticity, i.e. potentiation and depression, can be enhanced by novelty exploration [62, 70, 135], and the enhancement effects depend on the activation of β-adrenoceptors [55, 117].

Finally, these behavioral modulations by NA are closely related to stress and anxiety, since NA is known to be released during acute stressful episodes [87]. Following chronic stress, large cognitive impairments are observed, and they can be improved by antidepressants such as desipramine that blocks NA reuptake [15], suggesting down-regulation of NA transmission after chronic stress exposure.

## CONCLUDING REMARKS

NA participates in the modulation of a large spectrum of behaviors. NA appears to be related to the capacity of the organism to reach different levels of waking states, to integrate sensory information, and to engage in central cognitive processes such as memory. These different functions seem to be associated with the release of NA in various distinct areas.

From a cellular point of view, NA has been described to act as a “gating” agent [129-131]. This action consists of its capacity to change the threshold of postsynaptic neurons necessary to induce a sensory response. For example, iontophoretically applied NA induces a decrease in the discharge threshold in the majority of auditory cells and a global increase in the “signal-to-noise ratio” [71]. Another similar concept of NA action is “tuning”. NA participates in the selection of sensory responses to a specific class of sensory stimulation *via* inhibition of the response to adjacent classes of sensory stimuli [49, 72]. These effects were shown to last for more than 15 min [72] and were mediated by α-adrenoceptors [121]. NA’s ability to enhance the “signal-to-noise ratio” can be interpreted as a mechanism to select the most salient information within a neural network. Moreover, NA also modulates the integration of information by an improvement of spike timing precision [59]. These two last cellular actions of NA may be related to high cognitive functions such as the selection of pertinent behavior, which sometimes needs behavioral switches to flexibly meet contextual demands [16].

Considering the diverse actions induced by NA, the importance of the concentrations used should be pointed out [36]. Armstrong-James and Fox [7] found an inhibitory effect with an elevated quantity of NA whereas with a lower level, it exerted an excitatory effect on the spontaneous activity of somatosensory cortex. A similar effect was found in the hippocampus [83]. Globally, however, a general major action of NA in the brain seems to be inhibitory [35, 64, 106, 126, 127, 129], as we have observed in PFC synaptic transmission (Marzo *et al.*, unpublished data). But some biphasic actions are also described, where the excitatory effect of NA was transient and followed by an inhibition [16, 126].

NA effects may also depend on layers within a single structure. In somatosensory cortex, middle layer neuronal activity was decreased by NA in the majority of cases, whereas neurons in deep and superficial layers showed NA-induced excitation for both evoked and spontaneous activities [127]. Our laboratory also observed differential effects of NA in PFC slices where synaptic depression was observed in layer I-II to layer V pyramidal neuron synapses after NA application whereas layer VI synapses showed little change (Marzo *et al.*, unpublished data).

A recent trend in the plasticity field highlights the importance of neuroplasticity not only in physiological regulations but also in pathological regulations of brain networks. In this respect, we note that another catecholamine dopamine plays a major functional role in the PFC [39], through up- or down-regulation of PFC glutamatergic synapses depending on developmental and behavioral conditions. A main difference of the manner of dopaminergic regulation of PFC synaptic transmission from that of NA is that dopamine has to act temporally together with high-frequency conditioning input to glutamatergic synapses in order to exert its long-term modulatory effects on synaptic efficacy. NA, in contrast, depresses, or in some cases potentiates (Marzo *et al.*, unpublished data), PFC synapses without coincidental enhanced glutamatergic activity, suggesting global effects of NA on the signal-to-noise ratio. How these NA effects are related to behavior and whether there is any interaction between the dopamine-induced and NA-induced synaptic changes remain as important future questions.

In conclusion, the activation of noradrenergic system is able to induce and modulate intrinsic and synaptic plasticity in two different directions, depending on the concentration, the structure, and the engaged pathway. Further studies, giving more attention to these details, should reveal yet more specific links between behavior and NA modulation of brain networks.
